# Hydrocolpos causing bowel obstruction in a preterm newborn: a case report

**DOI:** 10.1186/s40748-024-00179-3

**Published:** 2024-05-02

**Authors:** Martin Jouza, Ingrid Rejdova, Lukas Cintula, Anna Jouzova, Petr Jabandziev

**Affiliations:** 1 Department of Pediatrics, University Hospital Brno, Cernopolni 9, 613 00 Brno, Czech Republic; 2 Faculty of Medicine, Masaryk University, Brno, Czech Republic; 3 Department of Obstetrics and Gynecology, University Hospital Brno, Brno, Czech Republic

**Keywords:** Hydrocolpos, Bowel obstruction, Preterm neonate, Imperforate hymen

## Abstract

**Background:**

Imperforate hymen is the most common congenital defect of the female urogenital tract. The spectrum of clinical manifestations is broad, ranging from mild cases undiagnosed until adolescence to severe cases of giant intraabdominal masses. The most common complication of hydrocolpos is bladder compression, resulting in obstructive uropathy and hydronephrosis.

**Case presentation:**

We present here the case of a preterm neonate who was admitted to the surgical neonatal intensive care unit for bowel obstruction. The baby did not appear septic or unwell, a small amount of meconium passed frequently, and no bilious gastric residuals occurred. Based on these findings, acute abdominal obstruction was doubtful, and the surgeon chose a conservative (watch and wait) approach. Subsequently, we performed abdominal ultrasound and magnetic resonance imaging based on unclear information about a suspicious abdominal mass raised by the gynecologist shortly before the emergency C-section. The final diagnosis was congenital hydrocolpos due to imperforate hymen. The pediatric gynecologist indicated an incision of the imperforate hymen under general anesthesia. The incision resolved abdominal distention as well as the bowel obstruction.

**Conclusion:**

The presentation of hydrocolpos was not typical (no bulging in the vaginal introitus) in our case, and clinical symptoms implied acute bowel obstruction shortly after birth. The surgeon chose a conservative (watch and wait) approach as the baby did not appear unwell on the second day of life. Fortunately, diagnostic laparotomy was not required as the next step in bowel obstruction management. All clinical symptoms resolved after a minor surgical procedure.

## Introduction

Imperforate hymen is the most common congenital defect of the female urogenital tract. It causes complete obturation of the vaginal introitus [1]. The incidence of congenital imperforate hymen varies in the literature between 0.0014% and 0.1% per year, whereas the incidence of hydrocolpos has been reported as 1 in 16,000 (i.e., 0.006%) term female newborns [2]. Congenital hydrocolpos is thus a rare event. Clinical manifestation is hydrometrocolpos in the newborn period or hematocolpos at the time of menstruation onset [1].

Hydrocolpos is defined as vaginal distention with fluid accumulation due to a combination of stimulated secretory glands and vaginal obstruction [3]. It is usually asymptomatic, and the average age of presentation or establishing a diagnosis is 11–15 years, with the onset of menstruation. Rarely is it diagnosed within the newborn period [4]. Hydrocolpos and imperforate hymen could also be associated with many other congenital anomalies of the urogenital tract, ranging from persistent urogenital sinus to cloacal dysgenesis [5].

## Case report

We present here the case of a preterm female newborn. The baby was delivered by emergency C-section for placental abruption at 33 + 4 gestational weeks to a G3P2 mother. Birth weight was 2490 g, and birth length 45 cm. Apgar score was 2–5–8. In the intermediate care unit, she needed ventilation support with nasal continuous positive airway pressure and the fraction of inspired oxygen up to 0.25 and with positive end-expiratory pressure of 7 cm H_2_O. Early feeding was initiated after delivery. However, abdominal distention, feeding refusal, and bilious gastric residuals were observed on the second day after birth. Abdominal distention remained, and she reacted painfully upon palpation. The septic screening was done without pathological findings. Parenteral nutrition was started, and a plain abdominal radiograph was made. It showed an absence of gas at the distal part of the colon. A follow-up radiograph after 12 h indicated persistent findings. At this point, the baby girl was transferred to the surgical neonatal intensive care unit (in a different part of the town). The baby was dyspneic with frequent apnea after the transportation. Therefore, we had to secure airways by orotracheal intubation. Nevertheless, the baby did not look unwell. The surgeon discovered slightly altered bowel sounds but no other pathological clinical findings (no distention, no abdominal masses). Even a small amount of meconium passed, and no more bilious gastric residuals occurred after the transportation. Based on these findings, volvulus seemed unlikely, and the surgeon chose a conservative (watch and wait) approach.

**Fig. 1:**
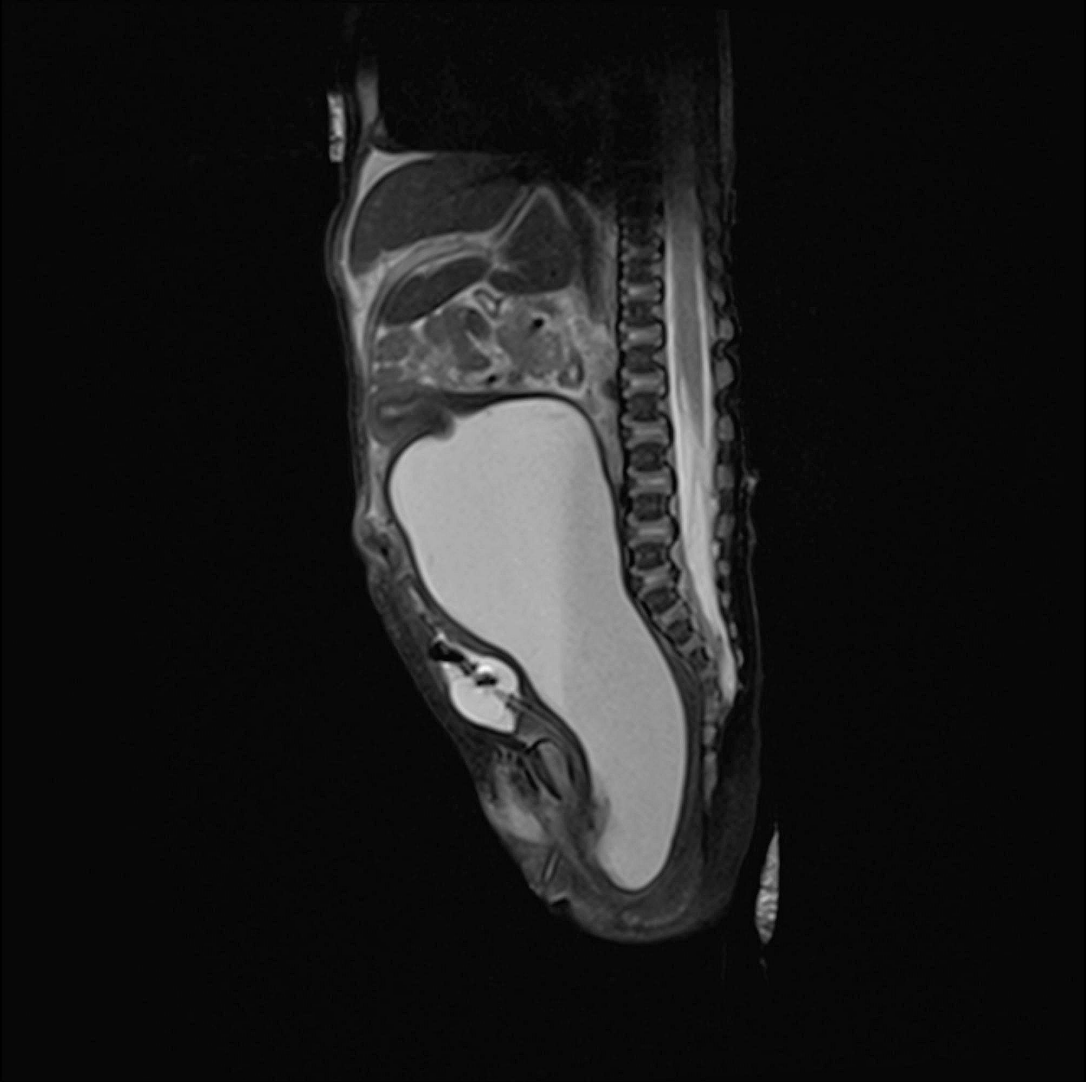
MRI of the abdomen and pelvis. Findings suggested a voluminous vagina with a fluid–fluid level extending from the perineum to the supraumbilical region

**Fig. 2:**
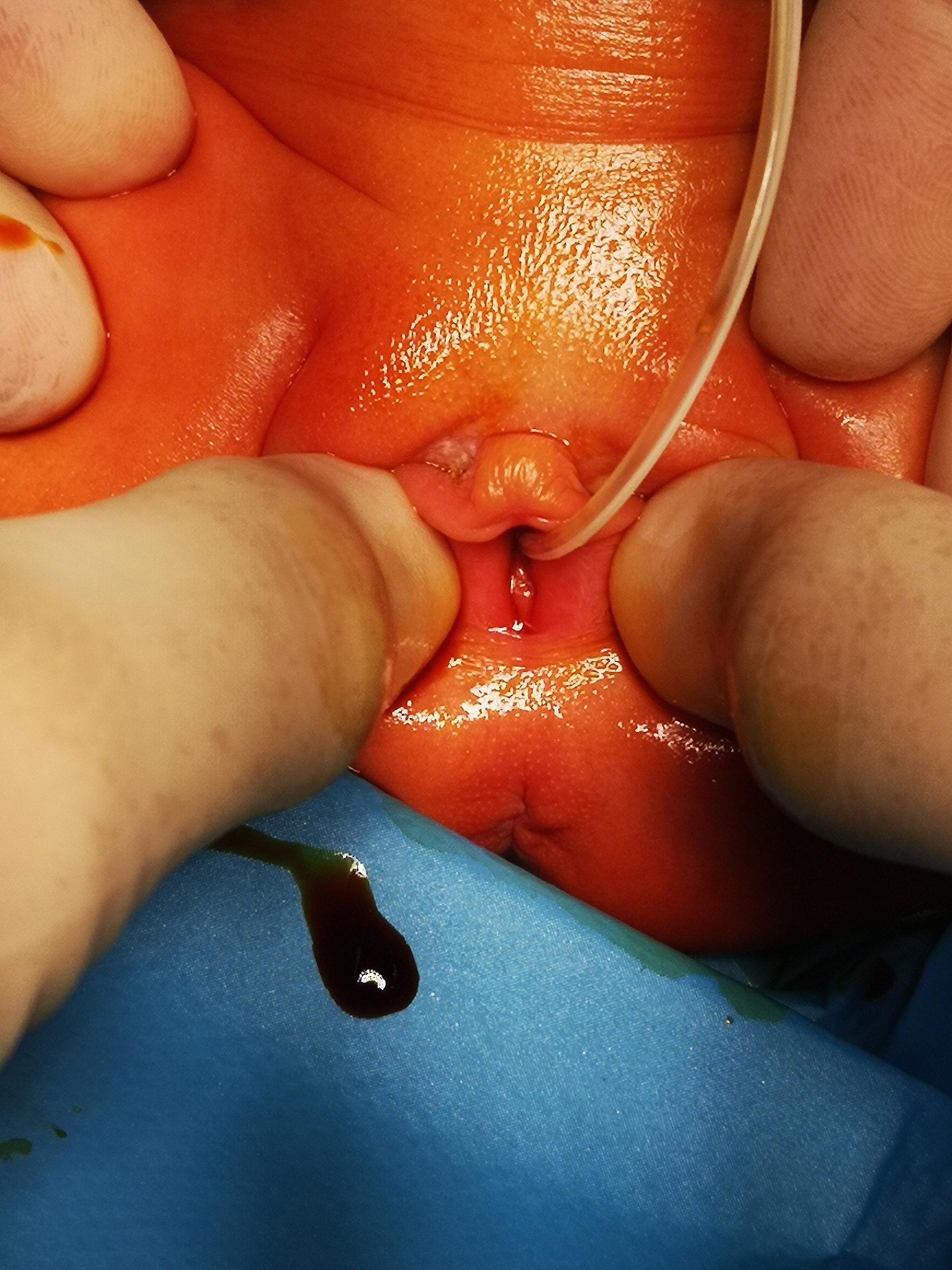
Physical examination revealed no bulging or other pathological findings at the vaginal opening

**Fig. 3:**
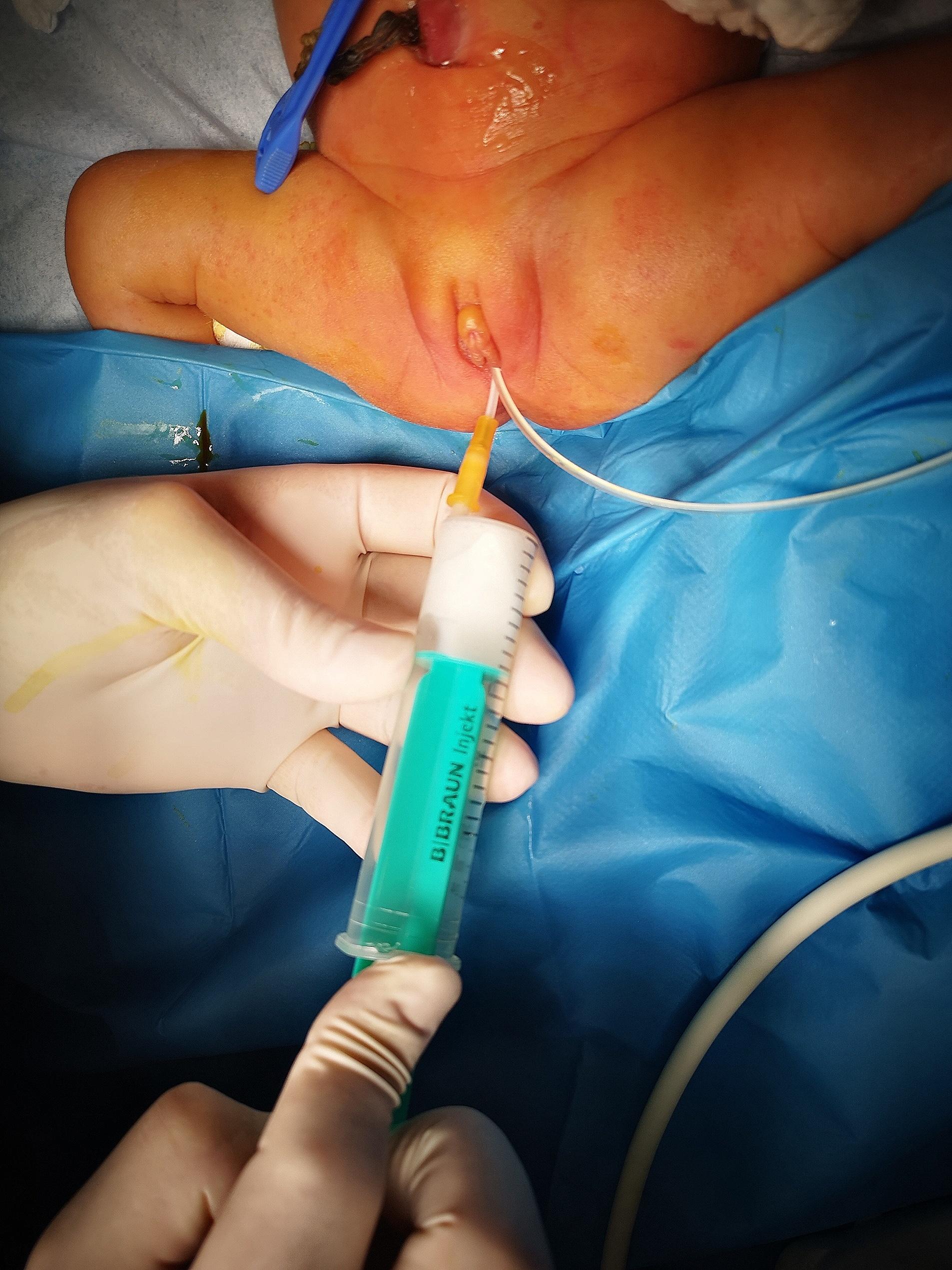
After the incision of the hymenal membrane, approximately 140 ml of milky fluid was aspirated

The new information about the suspicious intraabdominal mass has occurred. After a rash ultrasound examination shortly before birth, the gynecologist hypothesized a potential intraabdominal mass. The main finding was retroplacental hematoma needing the emergency C-section. The information about the suspicious intraabdominal mass was uncertain. Therefore, an abdominal ultrasound was performed on the second day of life. The radiologist described a large cystoid formation in the pelvic cavity from the perineum to the umbilical area, close behind the urinary bladder and ureter. We performed magnetic resonance imaging of the abdomen and pelvis (Fig. 1). Findings suggested a voluminous vagina with a fluid–fluid level extending from the perineum to the supraumbilical region. The final diagnosis was congenital hydrocolpos due to imperforate hymen, but a physical examination of the neonate revealed no bulging or other pathological findings at the vaginal opening (Fig. 2). Specific results were consulted with a pediatric gynecologist, and an incision was made of the imperforate hymen under general anesthesia. After the incision of the hymenal membrane, approximately 140 ml of milky fluid was aspirated (Fig. 3), and a drain was placed for three days. After drainage, abdominal distention and bowel obstruction were entirely resolved. No other congenital defects of the urinary tract were detected. During the admission complex, a congenital heart defect had been diagnosed. It was a ventricular septal defect, persistent left superior vena cava, and pulmonary sling. Considering all findings, genetic consultation and examination were recommended. Genetic testing detected no microdeletion of 22q11.2, but microduplication of 22q11.2 was found. After 27 days, the baby was discharged to home in good condition. She remains in cardiological, genetic, and gynecological follow-up.

## Discussion

Congenital imperforate hymen is the most frequently occurring congenital malformation of the female genital tract [6]. A comprehensive systematic review published in 2019 by Lee et al. encompassed 236 patients with imperforate hymen. Twenty-six subjects (11%) were in the neonatal period. Only 1 case had manifested with peritoneal signs, and there was not a single case with symptoms of bowel obstruction [7].

The differential diagnoses of intraabdominal masses in female newborns should include ovarian cyst, urachal cyst, ureterocele, meningocele anterior, mesenteric cyst, duplications of bowl, urinoma, sacrococcygeal teratoma, and liver cyst [8].

Although obstruction of vaginal introitus is most frequently caused by imperforate hymen, hydrocolpos could result from labial adhesion, transverse vaginal septum, vaginal atresia, and vaginal agenesis [9]. Complications of hydrocolpos, as described in the literature, could be connected with compression of the lower urinary tract, resulting in hydronephrosis and megaureters. As it may lead to renal failure, early detection of hydrocolpos is essential [7]. To our knowledge, ileus caused by hydrocolpos has never been described in the literature.

The clinical presentation of most such cases is bulging in the vaginal introitus. Therefore, close checking of the genitalia is essential to a newborn’s first examination. In our case, there was no bulging or any other pathological finding in the vaginal introitus, and the suggestion of intraabdominal mass had been established shortly before the emergency C-section. No abdominal mass had been noted at routine follow-ups during pregnancy.

The majority of such cases are treated surgically. The incision of an imperforate hymen and drainage of the fluid bring complete resolution of the fluid collection and resulting complications [2]. In our case, the incision of the hymen also resolved the bowel obstruction. The finding of microduplication of 22q11.2 could potentially affect the patient’s phenotype, but according to the literature, this finding is not connected to hydrocolpos or imperforate hymen [10].

## Conclusion

Although the prognosis after treating imperforate hymen is excellent, complications in the newborn period could be associated with high mortality and morbidity. Our patient was transferred to the surgical neonatal intensive care unit because of acute abdominal symptomatology indicating acute bowel obstruction of unknown etiology. The surgeon chose a conservative (watch and wait) approach, as the baby did not appear unwell on the second day of life. Prenatal diagnosis of hydrocolpos is crucial for immediate postnatal management. In our case, the information about the suspicious intraabdominal mass was very uncertain due to the emergency occurring before the C-section. In addition, there were no typical clinical presentations of a bulging in the vaginal introitus. Fortunately, we performed an abdominal ultrasound to manage bowel obstruction and established the diagnosis of hydrocolpos. Hence, diagnostic laparotomy was not required. All clinical symptoms resolved after a minor surgical procedure.

## Data Availability

All data are not publicly available. Further inquiries can be directed to the corresponding author.
